# Prevalence of Comorbidities and Their Impact on Hospital Management and Short-Term Outcomes in Vietnamese Patients Hospitalized with a First Acute Myocardial Infarction

**DOI:** 10.1371/journal.pone.0108998

**Published:** 2014-10-03

**Authors:** Hoa L. Nguyen, Quang Ngoc Nguyen, Duc Anh Ha, Dat Tuan Phan, Nguyen Hanh Nguyen, Robert J. Goldberg

**Affiliations:** 1 Institute of Population, Health and Development, Ha Noi, Viet Nam; 2 Oxford University Clinical Research Unit, Ho Chi Minh City, Viet Nam; 3 Viet Nam National Heart Institute, Ha Noi, Viet Nam; 4 Ministry of Health, Ha Noi, Viet Nam; 5 Department of Quantitative Health Sciences, University of Massachusetts Medical School, Worcester, Massachusetts, United States of America; Azienda Ospedaliero-Universitaria Careggi, Italy

## Abstract

**Background:**

Cardiovascular disease is one of the leading causes of morbidity and mortality in Vietnam. We conducted a pilot study of Hanoi residents hospitalized with a first acute myocardial infarction (AMI) at the Vietnam National Heart Institute in Hanoi for purposes of describing the prevalence of cardiovascular (CVD) and non-CVD comorbidities and their impact on hospital management, in-hospital clinical complications, and short-term mortality in these patients.

**Methods:**

The study population consisted of 302 Hanoi residents hospitalized with a first AMI at the largest tertiary care medical center in Hanoi in 2010.

**Results:**

The average age of study patients was 66 years and one third were women. The proportions of patients with none, any 1, and ≥ 2 CVD comorbidities were 34%, 42%, and 24%, respectively. Among the CVD comorbidities, hypertension was the most commonly reported (59%). There were decreasing trends in the proportion of patients who were treated with effective cardiac medications and coronary interventions as the number of CVD comorbidities increased. Patients with multiple CVD comorbidities tended to develop acute clinical complications and die at higher rates during hospitalization compared with patients with no CVD comorbidities (Odds Ratio: 1.40; 95% Confidence Interval: 0.40–4.84).

**Conclusions:**

Our data suggest that patients with multiple cardiac comorbidities tended to experience high in-hospital death rates in the setting of AMI. Full-scale surveillance of Hanoi residents hospitalized with AMI at all Hanoi hospitals is needed to confirm these findings. Effective strategies to manage Vietnamese patients hospitalized with AMI who have multiple comorbidities are warranted to improve their short-term prognosis.

## Introduction

The prevalence of persons with multiple comorbidities in the general population is increasing, even in low-middle income countries (LMICs) [1], [2], [3]. Data from the International Network for Demographic Evaluation of Populations and Their Health including 8 Asian sites in Vietnam, Bangladesh, Thailand, Indonesia, and India in 2005 showed that 23% of men and 32% of women aged 25–64 years reported having at least 1 chronic health condition, and 5% of men and 9% of women reported having 2 or more chronic conditions [2]. Patients hospitalized with acute myocardial infarction (AMI) also have a variety of cardiovascular (CVD) and non-CVD comorbidities present [4], [5], [6], [7], [8]. Previous studies have suggested that CVD and non-CVD comorbidities exert a negative impact on in-hospital complications and mortality in patients hospitalized with AMI, though these studies have been primarily conducted in high-income countries [4], [5], [6], [7], [8], [9].

Vietnam is an LMIC, which has been undergoing an important epidemiological transition. The overall morbidity and mortality from Non-Communicable Diseases (NCDs) has been rising rapidly over the last two decades and the NCDs have become a major societal problem. CVD is now the leading cause of death in Vietnam, accounting for approximately one quarter of all deaths annually. Moreover, the major risk factors for CVD are either on the rise or at disturbing levels in the Vietnamese population [10]. Data from the International Network for Demographic Evaluation of Populations and Their Health in 2005 showed that the prevalence of persons with 2 or more chronic conditions was approximately 6% for Vietnamese men and 7% for women [2].

Despite the important impact of multiple comorbidities on the prognosis of patients hospitalized with AMI, to the best of our knowledge no studies have reported the prevalence of CVD and non-CVD comorbidities and their impact on hospital management practices and short-term outcomes among adults hospitalized with AMI in Vietnam. The objectives of this observational study were to describe the prevalence of several CVD and non-CVD comorbidities and their impact on hospital management, in-hospital clinical complications, and mortality in patients hospitalized with a first AMI during 2010 at the Vietnam National Heart Institute in Hanoi.

## Methods

### Ethics Statement

The Institutional Review Board at the Institute of Population, Health and Development approved this study. The study was conducted with a waiver of patient consent approved by the institute's Institutional Review Board. Patient records/information was anonymized and de-identified prior to analysis.

### Study Setting

This study was carried out at the Vietnam National Heart Institute [11]. This facility is a 250 bed- tertiary care medical center in Hanoi (2009 census  = 6.5 million), which treats the majority of Hanoi residents hospitalized with acute coronary disease and other NCDs.

### Case Ascertainment Approaches

Computerized printouts of patients discharged from the Vietnam National Heart Institute in 2010 with possible AMI were obtained and International Classification of Disease codes for possible AMI (I20-I25) were reviewed. Cases of possible AMI were independently validated according to predefined criteria for AMI by two cardiologists. The diagnosis of AMI was made on the basis of criteria developed by the World Health Organization which includes a suggestive clinical history, serum enzyme elevations above the hospital's normal range (normal ranges: Troponin I<0.5 ng/ml, Troponin T<0.01 ng/ml, CK-MB <7–25 U/L, CK: Male: 38–174 U/L, Female: 26–140 U/L), and serial electrocardiographic (ECG) findings during hospitalization consistent with the presence of AMI; at least 2 of these 3 criteria needed to be present for an AMI to have occurred [12]. A total of 315 residents of the city of Hanoi who satisfied criteria for the diagnosis of AMI were identified. Since the majority (302 patients) of this population was hospitalized for a first AMI (96%), we restricted our patient population to those with an initial AMI based on the review of information obtained from hospital medical records. Our patient population was also further classified into those with ST segment elevation AMI (STEMI) and non-ST segment elevation AMI (NSTEMI), using standard classification techniques [13]. Each of the ECG's of patients with possible AMI was reviewed by a physician under the guidance of a senior cardiologist. A small number of patients with AMI (n = 21) were not able to be classified as STEMI or NSTEMI due to the poor quality of reviewed ECGs.

### Data Collection

We reviewed the paper medical records of all patients who were admitted with discharge diagnoses suggestive of AMI and reviewed each of their hospital charts. For each independently validated case of AMI who satisfied our geographic eligibility criteria, a variety of socio-demographic, clinical, and medical care related information was collected from the review of the medical record and abstracted onto a standardized case-report form by trained study physicians. Information was collected about patient's age, sex, previously diagnosed comorbidities, admission heart rate, blood pressure, serum glucose and serum lipid levels, AMI type (STEMI vs. NSTEMI), receipt of effective cardiac medications during the index hospitalization (e.g., Angiotensin converting enzyme inhibitors-ACEIs; Angiotensin II receptor blockers-ARBs, beta blockers) and coronary interventional procedures (e.g., percutaneous coronary intervention -PCI, coronary artery bypass surgery -CABG), clinically significant in-hospital complications (e.g., heart failure, stroke, atrial fibrillation), hospital discharge status, and length of hospital stay.

Comorbidities in the present study were defined as those chronic conditions that were previously diagnosed, and had been documented, in the medical history section of reviewed hospital charts, and did not include any conditions that may have been newly diagnosed during the patient's hospital admission. Thus, there were no clinical or laboratory criteria that were used to make the final diagnoses for these conditions. Comorbidities in this patient population were categorized into 2 groups: CVD and non-CVD comorbidities. Seven CVD comorbidities were considered including atrial fibrillation, coronary heart disease -CHD (prior angina, CHD, MI, CABG, and PCI), diabetes, heart failure, hypertension, hyperlipidermia, and stroke. Six non-CVD comorbidities including anemia, asthma, cancer, chronic obstructive pulmonary disease (COPD), gastro-intestinal (GI) bleeding/peptic ulcer, and chronic renal disease were examined. The selection of these conditions was made based on findings from previous studies that have examined the associations of these comorbdiities with the prognosis of patients hospitalized with AMI [4], [5], [6], [7], [8] and based on their prevalence in this patient sample.

The primary outcome of the present study was the in-hospital case- fatality rate (CFR). Secondary outcomes included hospital management practices (e.g., use of aspirin, ACEIs/ARBs, beta blockers, statins, PCI, and CABG), hospital clinical complications (e.g., atrial fibrillation, heart failure, cardiogenic shock), and hospital length of stay. In terms of hospital clinical complications, atrial fibrillation included the documentation of new atrial fibrillation in the hospital medical record or occurrence of typical electrocardiographic changes consistent with this diagnosis. Heart failure was indicated by clinical or radiographic evidence of pulmonary edema or bilateral basilar rales with an S3 gallop. Cardiogenic shock was defined as a systolic blood pressure of less than 90 mmHg in the absence of hypovolemia and associated with cyanosis, cold extremities, changes in mental status, persistent oliguria, or congestive heart failure.

### Data Analysis

The overall prevalence of single and multiple comorbidities in this population was calculated in a standard manner. The number of CVD comorbidities was categorized into 3 categories (0, 1, and ≥2) and the number of non-CVD comorbidites was categorized into 2 categories (0 and ≥1). Data were presented as percentages for categorical variables and compared using the chi-square or Fisher exact tests if expected values were less than 5; medians (inter quartile range- IQR) for continuous variables were calculated and compared using the Kruskal-Wallis tests.

Logistic regression models were used to examine the impact of number of comorbidities on in-hospital CFRs. A multivariable logistic regression model was adjusted for several patient associated characteristics that significantly differed according to number of comorbidities in unadjusted analyses (age, MI characteristic: STEMI vs. NSTEMI). The most common clusters of co-existing comorbidities were identified and in-hospital CFRs associated with these clusters were reported. Two-sided analytic tests were used and a p value less than 0.05 was considered to be statistically significant. All analyses were performed using STATA 11.0 (StataCorp. TX).

## Results

The study population consisted of 302 Hanoi residents hospitalized with a first AMI at the VNHI in 2010. The average age of study patients was 66 years and one third were women. Seventy three percent of patients developed an STEMI. The overall in-hospital mortality was 7.0%.

### Prevalence of CVD and Non-CVD Comorbidities

**Figure 1:**
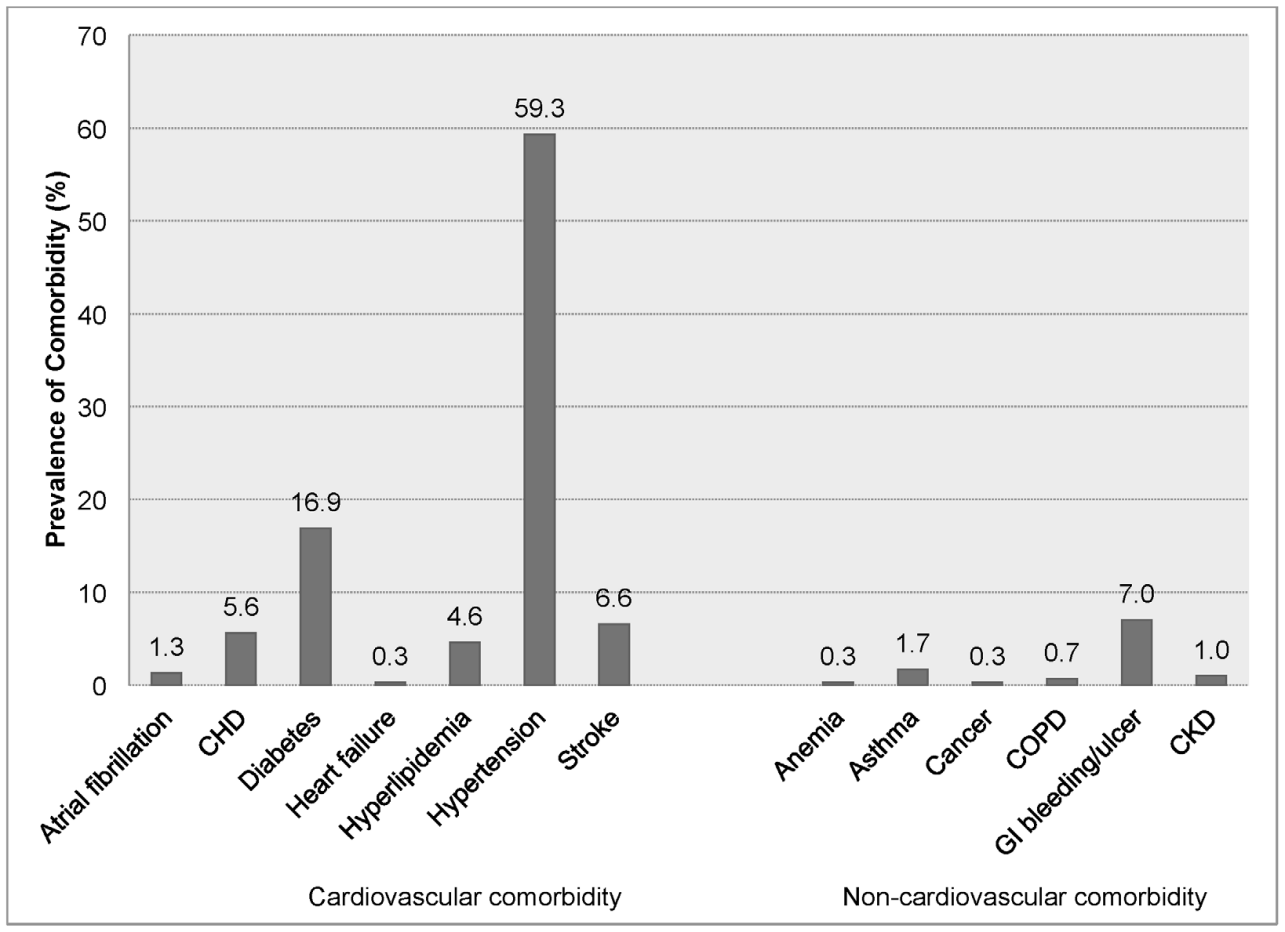
Prevalence of Selected Cardiovascular and Non-Cardiovascular Comorbidities among Patients Hospitalized with Acute Myocardial Infarction. CHD, coronary heart disease; COPD, chronic obstructive pulmonary disease; GI, gastro-intestinal; CKD, chronic kidney disease.

The mean and median number of CVD comorbidities in this population were 0.9 and 1 respectively (range: 0-3; only 2 patients had 3 conditions). Two thirds of patients had at least 1 CVD comorbidity previously diagnosed. The proportions of patients with none, any 1, and any 2 or more CVD comorbidities were 34%, 42%, and 24%, respectively. Among the cardiac comorbid conditions examined, hypertension was the most commonly reported (59%) followed by diabetes (17%) (Figure 1).

The majority (90%) of patients had none of the non-CVD comorbidities examined and only 10% had any 1 or more non-CVD comorbidities previously diagnosed. Among the non-CVD comorbidities, GI bleeding/ulcer was the most commonly reported (7%) (Figure 1). Since the prevalence of non-CVD comorbidities was very low in this population, we did not perform further analyses according to the number of non-CVD comorbidities present.

### Study Population Characteristics

Patients with any CVD comorbidity were significantly older and more likely to have developed a NSTEMI than patients without any previously diagnosed CVD comorbidity (Table 1). On admission, patients with any CVD comorbidity had significantly higher blood pressure results, higher plasma glucose levels, but lower estimated glomerular filtration rate (eGFR) findings compared to patients without any CVD comorbidity.

**Table 1:** Study Population Characteristics According to Number of Cardiovascular Comorbidities.

	0 (n = 103)	1 (n = 127)	≥2 (n = 72)	p value*
Age (mean, SD), years	63(13)	68(11)	67(10)	0.003
Male	76(73.8)	83(65.4)	42(58.3)	0.10
Ethnicity (n,%)				
Kinh**	100(97.1)	127(100)	69(95.8)	0.09
Minority	3(2.9)	0(0)	3(4.2)	
MI characteristic (n,%)				
STEMI¶	80(83.3)	84(72.4)	43(62.3)	0.010
Non-STEMI	16(16.7)	32(27.6)	26(37.7)	
Clinical parameters on admission (median, IQR)				
Heart rate (beats/min)	82(72–97)	87(72–102)	90(79–102)	0.16
Systolic blood pressure (mmHg)	120(100–130)	120(110–145)	130(120–140)	0.002
Diastolic blood pressure (mmHg)	70(60–80)	80(70–90)	80(70–90)	0.008
Laboratory findings on admission (median, IQR)				
Cholesterol (mmol/L)	4.5(3.9–5.0)	4.6(4.0–5.1)	4.4(4.0–5.3)	0.84
LDL (mmol/L)	2.7(2.1–3.1)	2.8(2.3–3.1)	2.6(2.2–3.4)	0.71
Glucose (mmol/L)	6.8(5.4–9.0)	7.5(5.7–9.0)	8.5(6.1–12.2)	0.008
eGFR (ml/min/1.73 m^2^)°	75(62–92)	67(52–80)	68(54–89)	0.002
Cardiovascular comorbidity (n,%)				
Atrial fibrillation	NA	2(1.6)	2(2.8)	
Coronary heart disease	NA	4(3.1)	13(18.1)	
Diabetes	NA	10(7.9)	41(56.9)	
Heart failure	NA	0(0)	1(1.4)	
Hypertension	NA	108(85)	71(98.6)	
Hyperlipidemia	NA	1(0.8)	13(18.1)	
Stroke	NA	2(1.6)	18(25)	

SD: Standard deviation; IQR: Inter quartile range; STEMI: ST segment elevation AMI; LDL: Low Density Lipoprotein

**The Kinh people are the majority ethnic group in Vietnam, comprising 87% of the population (census 2009)

¶ data missing in 21 patients

°eGFR: glomerular filtration rate was calculated based on CKD-EPI Equation

*p values from chi-square or Fisher exact tests for categorical variables and Kruskal-Wallis tests for continuous variables.

### Hospital Management Practices

Overall, during the first 24 hours after hospital admission, the use of aspirin and lipid lowering agents was high (>90%) as was the use of ACE inhibitors (∼70%); however, the use of beta-blockers was comparatively low (∼30%) (Table 2).

**Table 2:** Study Outcomes According to Number of Cardiovascular Comorbidities.

	0 (n = 103)	1 (n = 127)	≥2 (n = 72)	p value*
Management in the first 24 hours after admission (n,%)				
Aspirin	100(97.1)	123(96.9)	65(90.3)	0.06
ACE Is	73(70.9)	91(71.7)	51(70.8)	0.99
Beta blockers	26(25.2)	25(19.7)	11(15.3)	0.26
Lipid lower agents	96(94.1)	118(92.9)	62(86.1)	0.14
All 4 medications	23(22.3)	22(17.3)	10(13.9)	0.34
Percutaneous coronary intervention	64(62.7)	74(58.3)	38(52.8)	0.42
Management during hospitalization (n,%)				
Aspirin	102(99.0)	127(100)	71(98.6)	0.46
ACE Is/ARB	86(83.5)	111(87.4)	58(80.6)	0.42
Beta blockers	34(33.0)	41(32.3)	16(22.2)	0.24
Lipid lower agents	97(94.2)	118(92.9)	63(87.5)	0.25
All 4 medications	30(29.1)	36(28.3)	16(22.2)	0.56
Hospital cardiac procedures (n,%)				
Cardiac catheterization	84(81.6)	95(74.8)	54(75.0)	0.42
Percutaneous coronary intervention	73(70.9)	87(68.5)	47(65.3)	0.74
Coronary artery bypass surgery	3(3.0)	1(0.8)	0(0)	0.36
Hospital complications (n,%)				
Atrial fibrillation	2(1.9)	2(1.6)	0(0)	0.68
Cardiogenic shock	5(4.9)	5(3.9)	2(2.8)	0.87
Heart failure	11(10.7)	15(11.8)	13(18.1)	0.32
Recurrent angina	4(3.9)	6(4.7)	5(6.9)	0.65
Stroke	0(0)	1(0.8)	0(0)	0.99
Ventricular fibrillation or cardiac arrest	7(6.8)	6(4.7)	3(4.2)	0.76
In-hospital case-fatality rates (n,%)	7(6.8)	7(5.5)	7(9.7)	0.53
Length of hospital stay (median, IQR), days	4(3–7)	4(2–5)	4(2–6)	0.17

ACE-Is: Angiotensin converting enzyme inhibitors; ARBs: Angiotensin II receptor blockers

*p values from chi-square or Fisher exact tests for categorical variables and Kruskal-Wallis tests for continuous variables.

During the first 24 hours after hospital admission, although there were no statistically significant differences in medication utilization both singly and in combination in relation to the number of CVD comorbidities previously diagnosed, there was a decreasing trend in the proportion of patients who received effective cardiac medications as the number of cardiac comorbidities increased (Table 2). Similarly, the proportion of patients undergoing PCI tended to decrease with increasing number of CVD comorbidities present (63% to 53%). Similar findings were observed when we examined the use of medications and cardiac procedures during the entire hospitalization.

### In-Hospital Clinical Complications

During hospitalization, although there were no statistically significant differences with regards to the risk of developing important clinical complications in relation to the number of CVD comorbidities, there was an increasing trend in the development of heart failure (from 11% to 18%), and recurrent angina (4% to 7%) as the number of CVD comorbidities increased (Table 2). However, there was a decreased likelihood of developing cardiogenic shock (5% to 3%) and ventricular fibrillation/cardiac arrest (7% to 4%) as the number of CVD comorbidities increased.

During hospitalization, although there were no statistically significant differences in terms of the risk of dying according to the number of CVD comorbidities, the crude in-hospital CFRs tended to be lower in patients with any 1 CVD comorbidity compared to those without any CVD comorbidity (5.5% vs. 6.8%, Table 2). After adjusting for age and AMI associated characteristic (STEMI vs. NSTEMI), there was a 29% lower risk of dying for patients with any 1 cardiac condition compared with patients without any CVD condition (adjusted OR: 0.71; 95%CI: 0.22–2.31).

On the other hand, the crude in-hospital CFR tended to be higher in patients with 2 or more CVD conditions compared to those without any CVD condition (9.7% vs. 6.8%, Table 2). After adjusting for age and AMI related characteristic, patients with multiple CVD comorbidities were 40% more likely to have died during hospitalization compared to patients without any cardiac condition (adjusted OR: 1.40; 95%CI: 0.40–4.84). Hospital length of stay did not differ according to the number of CVD comorbidities previously diagnosed.

### Common Clusters of CVD Comorbidities

The most common clusters of CVD comorbidities included “hypertension + diabetes” (n = 40,13.3%), “hypertension + stroke” (n = 18, 6.0%), “hypertension + CHD” (n = 12, 4.0%), and “hypertension + hyperlipidemia” (n = 13, 4.3%); the in-hospital CFRs for these clusters were 5.0%, 9.5%, 25.0% and 7.7%, respectively. Hypertension was included in all common clusters, and the in-hospital CFR for patients with both hypertension and CHD conditions was the highest, being approximately 3 times as high as that of the whole study population (overall CFR of 7.0%).

## Discussion

The results of our pilot study in Hanoi, Vietnam showed that approximately two thirds of patients hospitalized with a first AMI at the largest tertiary care center in Hanoi had at least 1 CVD comorbidity present. In terms of non-CVD comorbidity, the majority of our study sample had none, and only a tenth had any 1 or more of these conditions. Patients with multiple CVD comorbidities were more likely to be older, to have developed a NSTEMI, and tended to be at higher risk for dying during hospitalization compared with patients without any CVD comorbidity.

### Prevalence of CVD and Non-CVD Comorbidities

Our study showed that nearly one half of patients hospitalized with a first AMI had any 1, and a quarter had any 2 or more, CVD comorbidities; only one tenth of the study population had at least 1 non-CVD comorbidity previously diagnosed. Both the average number of comorbidities, and prevalence of multiple comorbidities, in our study population were lower than those reported in studies from high-income countries [4], [5], [6], [7], [8], [9] including Spain, Denmark, the United States and Switzerland. These differences may be explained partially by the fact that our study population was relatively younger and included only patients hospitalized with their first AMI. However, given the aging Vietnamese population, and the increasing adoption of a western style diet and various lifestyle practices, it is expected that the prevalence of patients with multiple chronic conditions will continue to increase in Vietnam. Inasmuch, monitoring trends in the frequency of individual and multiple comorbidities among patients hospitalized with AMI will be of continued importance to inform health systems and practitioners to design and provide more optimal patient care for these complex patients.

In our study, hypertension was found to be the most prevalent cardiac condition followed by diabetes. These findings are consistent with the results of other studies in LMICs [14], [15], [16], [17] and in high-income countries [4], [5], [6], [7], [8] where the prevalence of hypertension has ranged from approximately 50%–75%, and the prevalence of diabetes has ranged from 17%–46% in patients hospitalized with AMI. However, the prevalence of hyperlipidemia in our study was relatively low (5%) compared to that of other LMICs where the prevalence has ranged from 15–30% [14], [15], [16], [17]. GI bleeding/ulcer was found to be the most common non-CVD comorbidity in our patient population and other conditions were found at extremely low rates. These findings are in contrast to those observed in high-income countries [4], [7], [8] where chronic kidney disease, COPD, and depression have been noted in a significant proportion of these diverse study populations. For example, data from more than 30,000 patients with an acute coronary syndrome in the nationwide AMIS Plus registry in Switzerland between 2002–2012 showed that 7%, 6%, and 2% of patients reported having kidney disease, chronic lung disease, and a GI ulcer, respectively [9]. Similarly, in a study of more than 5,000 patients hospitalized with AMI in Spain between 2003–2009, the prevalence rates of kidney disease, chronic lung disease, depression, and GI ulcer were 11%, 16%, 4%, and 0.5%, respectively, in 2009 [4]. Previous studies from LMICs [14], [15], [16], [17] have failed to report the frequency of non-CVD comorbidities, so we were unable to compare our findings with the data from other LMICs.

### Factors Associated with Multiple Comorbidities

In our study population, patients with multiple cardiac comorbidities were more likely to be older, to have developed a NSTEMI, and to have higher serum glucose, but lower eGFR findings, compared with patients without any cardiac comorbidity. This finding is consistent with previous studies in the setting of AMI [4], [5], [6], [7], [8], [9], as well as studies in general population samples from LMICs [1], [2], [3], [18]. For example, in the Worcester Heart Attack Study of nearly 3,000 patients hospitalized with AMI in central Massachusetts (United States) between 2003–2007, patients with multiple chronic conditions were more likely to be older, female, to have developed an NSTEMI, and to have higher serum glucose, but lower eGFR and serum cholesterol levels, at the time of hospital admission compared to those without any cardiac comorbidity [8]. Identifying patient characteristics associated with multiple cardiac comorbidities is important to inform interventions designed to decrease the prevalence of multiple comorbidities in these individuals.

### Hospital Management Practices

Although there were no statistically significant differences in hospital management practices in relation to the number of chronic conditions previously diagnosed in the present study, our results suggest that there was a decreasing trend in the receipt of effective cardiac medications and PCI as the number of cardiac comorbidities increased, consistent with the findings from several developed countries [7], [8], [9]. For example, a study of nearly 3,000 patients with AMI in 53 hospitals in Ontario, Canada between 1999–2003 demonstrated that the number of pre-existing conditions was inversely associated with the receipt of coronary reperfusion therapy [7]. Similarly, data from the AMIS Plus registry found that patients with multiple (CVD and non-CVD) comorbidities were less likely to have received effective cardiac medications and coronary reperfusion therapy compared to patients with no comorbidity [9].

Patients with multiple comorbidities are challenging to treat due to issues related to possible medication contraindications and their high rates of adverse events. Current management guidelines in Vietnam focus only on patients with a single condition and not on those with multiple conditions. Thus, developing comprehensive management strategies for these patients is urgently needed. One such approach to consider would be a patient-and family centered approach to care throughout the health system [19].

### In-hospital Complications and Death Rates

Our data showed that as the number of CVD comorbidities increased, the risk of developing heart failure and cardiogenic shock increased, as did the number of patients who died during hospitalization. These findings are consistent with previous studies [4], [5], [8], [9]. For example, a nationwide study in Denmark including more than 200,000 patients hospitalized with a first AMI between 1984–2008 found that patients with multiple comorbidities experienced higher short- and long-term mortality compared to patients without multiple comorbidities [5].

In the present study, we also found that the risk of ventricular fibrillation or cardiac arrest tended to decrease as the number of CVD comorbidities increased. Similar findings have been reported in patients with other conditions, such as heart failure, in which the risk of non-sudden death and total mortality increased as the number of comorbidities increased, whereas the risk of sudden death/ventricular fibrillation decreased [20].

There are several potential explanations for these findings. First, patients with multiple comorbidities tend to be older and female, and older age and female sex have been found to be independent risk factors for mortality in patients with AMI [21], [22], [23], [24]. Second, patients with multiple comorbidities may misinterpret the symptoms of AMI and delay seeking timely medical care. Furthermore, patients with multiple comorbidities were less likely to be treated with effective medications and coronary interventions that may increase their risk of dying in hospital [7], [8], [9]. Finally, patients with multiple comorbidities tend to develop clinical complications that can contribute to higher mortality. More educational approaches and interventional efforts targeted at patients with multiple comorbidities are needed to improve their hospital outcomes.

### Common Clusters of Multiple CVD Comorbidities

Our study found that the most common clusters of chronic CVD comorbidities were “hypertension and diabetes”, “hypertension and stroke”, “hypertension and CHD”, and “hypertension and hyperlipidemia”. These findings are in line with the results of other studies in patients with AMI from high-income countries [8], as well as data from general population samples [25]. Identifying the common patterns of chronic comorbidities in patients hospitalized with AMI will help in the design of effective management strategies in these difficult to treat patients.

### Study Strengths and Limitations

This study is the first to have examined the prevalence of CVD and non-CVD comorbidities and their impact on short-term outcomes in patients hospitalized with AMI in Vietnam. The study has several limitations that must be kept in mind in interpreting our study results, however. First, since the population included only patients hospitalized at one hospital, the Vietnam National Heart Institute in Hanoi, the generalizability of our findings to patients admitted to other hospitals in Hanoi is limited and unknown. Since the sample size in the present study was small, we were underpowered to detect meaningful differences in other patient characteristics, hospital management practices, and short-term outcomes. Third, due to the nature of the study's retrospective design, we likely underestimated the prevalence of comorbidities in our study population. We also did not have information on several patient-associated characteristics (e.g., socioeconomic status, psychological factors, body mass index) which may have confounded some of the observed associations [1], [3]. We had very limited information on medication contra-indications; therefore, we were unable to carry out an analysis restricted to patients without contraindications to the medications examined, and likely underestimated the use of appropriate medications. Finally, because patients who died before hospitalization for AMI were not included, our findings are only generalizable to patients hospitalized with AMI.

## Conclusions

In summary, our findings highlight the importance of identifying patients hospitalized with AMI who have multiple CVD comorbidities to provide better care to these high risk and complex patients, and therefore, improve their short-term prognosis. Continued monitoring of trends in multiple comorbidities in patients hospitalized with AMI and other conditions of major public health importance, such as stroke, cancer, and chronic lung disease, in Vietnam remains of considerable clinical and public health importance.
